# A ribodepletion and tagging protocol to multiplex samples for RNA-seq based virus detection: application to the cassava virome

**DOI:** 10.1186/s12985-025-02634-9

**Published:** 2025-02-05

**Authors:** Daniel H. Otron, Justin S. Pita, Murielle Hoareau, Fidèle Tiendrébéogo, Jean-Michel Lett, Pierre Lefeuvre

**Affiliations:** 1 The Central and West African Virus Epidemiology (WAVE) for Food Security Program, Pôle Scientifique Et d’Innovation, Université Félix Houphouët-Boigny (UFHB), Abidjan, 22 BP 582 Côte d’Ivoire; 2 CIRAD, UMR PVBMT F-97410, St Pierre, La Réunion, France; 3 UFR Biosciences, Université Félix Houphouët-Boigny (UFHB), Abidjan, 22 BP 582 Côte d’Ivoire; 4 CIRAD, UMR PVBMT, Department of Plant Protection, College of Agriculture, Can Tho University, Can Tho City, Vietnam

**Keywords:** Cassava, Virome, Ribodepletion, RNaseH, Multiplexing, High-throughput sequencing, Virus discovery

## Abstract

**Background:**

Cassava (*Manihot esculenta*, Crantz), is a staple food and the main source of calories for many populations in Africa, but the plant is beset by several damaging viruses. So far, eight families of virus infecting cassava have been identified; the *Geminiviridae* (ssDNA viruses responsible for cassava mosaic disease, CMD) and *Potyviridae* (ssRNA + viruses responsible for cassava brown streak disease, CBSD) families being the most damaging to cassava in Africa. In several cassava-growing regions, the co-existence of species and strains from these two families results in a complex epidemiological situation making it difficult to correctly identify the viruses in circulation and delaying the implementation of disease management schemes. Nevertheless, the development of next generation sequencing (NGS) methods has revolutionized plant virus detection and identification. One NGS method that has been successfully used in virus detection and identification is ribodepleted RNA sequencing. Unfortunately, the relatively high cost makes it difficult to upscale this method to large epidemiological surveys and limits its adoption as a diagnostic tool.

**Results:**

Here, we develop a high-throughput sequencing protocol, named Ribo-M-Seq, that combines plant rRNA ribodepletion, cDNA synthesis, tagging with a 96 multiplexing scheme and Illumina sequencing. We evaluated the protocol on a series of cassava samples with a known assemblage of viruses. After confirming that the protocol was suitable for ribodepletion, we demonstrated it was possible to detect RNA and DNA viruses via identification of near full-size genomes. Additional phylogenetic analyses confirmed the presence of begomoviruses and ipomoviruses responsible for CMD and CBSD, respectively. We also detected a recently described ampelovirus (Manihot esculenta-associated virus) that was not detected in previous analyses.

**Conclusions:**

The use of the Ribo-M-Seq protocol will pave the way for large-scale sample analyses of collections with potentially complex viromes, such as those collected in the West African cassava integrated pest management program.

**Supplementary Information:**

The online version contains supplementary material available at 10.1186/s12985-025-02634-9.

## Background

Cassava (*Manihot esculenta*, Crantz) is the world’s fourth-largest source of calories after rice, wheat, and maize but, most importantly, is a staple food for around 800 million people globally [1, 2]⁠. Cassava cultivation is threatened by several diseases that cause severe yield loss [3]. In cassava-growing regions of Africa, cassava mosaic disease (CMD) and cassava brown streak disease (CBSD) are the main viral diseases causing yield loss, ranging from 40 to 100% [4, 5]. These two diseases are caused by a complex of eleven species of *Begomovirus* (ssDNA virus) [6]⁠ from the *Geminiviridae* family (*Cressdnaviricota* phylum), and two distinct species of *Ipomovirus* (ssRNA + viruses), cassava brown streak virus and Uganda cassava brown streak virus [7]⁠ from the *Potyviridae* family (*Pisuviricota* phylum), respectively. Recent studies have shown that CMD is present in all cassava-growing regions in Sub-Saharan Africa and Southern Asia. CBSD has been identified in East and Central Africa and the Comoros Archipelago [3], but is progressing towards West Africa despite control measures [8]. In addition, other viruses with a lesser or unknown impact [9, 10]⁠ have been identified, including one *Anulavirus* species (cassava Ivorian bacilliform virus) from the *Bromoviridae* family (ssRNA + virus, *Kitrinoviricota* phylum) [10]⁠ and two *Ampelovirus* species (Manihot esculenta-associated ampelovirus 1 and Manihot esculenta-associated ampelovirus 2) from the *Closteroviridae* family (ssRNA + viruses; *Kitrinoviricota* phylum) [9].

The prevention and management of plant viral diseases largely depends on the accurate identification of the viral communities responsible for the disease. However, the coexistence of several species and viral strains of these different viruses hampers the identification of circulating viruses. The absence of any canonical marker, such as the 16S gene for bacteria [11], has led virologists to develop approaches to enrich nucleic acid extracts with viral nucleic acids prior to sequencing. These next generation sequencing (NGS) methods have proved useful for the study and characterization of viromes from different sample types [12–15]. The most common approaches are virion-associated nucleic acids (VANA), double-stranded RNA (dsRNA), small interfering RNA (siRNA) and ribosomal RNA depleted total RNA [16, 17] sequencing. The latter is a credible alternative for virome characterisation and has been proved useful for the detection and discovery of RNA viruses, DNA viruses, and viroids [18, 19].

However, its use remains costly with, beside the cost of sequencing itself, costs associated to per-sample ribodepletion and sequencing library construction. Among the methods for rRNA depletion [20]⁠, RNaseH-mediated depletion (after the hybridization of reverse complementary specific DNA oligomers with rRNA, the resulting rRNA:DNA hybrids are cleaved with RNaseH endonuclease) has been proved efficient [21]. However, this procedure is mainly implemented using high price commercial kits that limits its large-scale use in many laboratories. A second large share of the global cost of the ribosomal RNA depleted total RNA sequencing is associated with library construction, with usually one library required for one sample. Whereas methodologies exist to analyse bulk samples [22], it then requires post hoc testing to trace back identified viruses to individual samples.

The aim of this study was to implement a cost-effective high-throughput sequencing approach devised for research purpose that combine ribodepletion of total RNA extracts and molecular tagging of nucleic acids for sample multiplexing before library construction and sequencing. Here, we propose the Ribo-M-Seq protocol, a high-throughput sequencing protocol based on the ribodepletion of total RNA, cDNA synthesis and tagging of individual samples before the pooling of bulk tagged cDNAs and Illumina sequencing. We tested the effectiveness of the RNaseH enzyme for rRNA depletion and virus characterisation on cassava samples with known viral populations. We found that ribodepletion by RNaseH efficiently depleted ribosomal RNA from cassava total RNA. We were able to multiplex samples, identify DNA and RNA viruses, and obtain near-complete genomes of the target viruses. Although tested on cassava, this metagenomic protocol for virome analysis can be adapted to other plants of agronomic or historic interest whose rRNA sequences are known.

## Methods

### Plant samples and virus infection status

Five virus-infected dried cassava leaf samples were used as virus-infected controls (Table 1). Samples were tested for their infection status using several approaches: double-stranded RNA (dsRNA) high-throughput sequencing [9]⁠ or PCR [23]⁠ or RT-PCR [24]⁠ followed by direct Sanger sequencing of amplicons. The infection status of each sample is described in Table 1. These five samples were collected in Comoros, Madagascar, Mayotte and Reunion between 2011 and 2016. Cassava leaves from uninfected vitroplants, frozen at −80 °C, were used as negative control.

**Table 1:** List of cassava samples used in the study with details on previous virus detections

Samples	Origin/Site	Collection date	Virus name (Acronyme)	Taxonomic name	Genome	Family/Genus	Virus detection	References
Healthy vitroplant	Reunion Island/ Saint-Pierre	20/01/2022	–	–	–	–	–	–
293MG040711	Madagascar/Diana	04/07/2011	African cassava mosaic virus (ACMV)	*Begomovirus manihotis*	ssDNA	*Geminiviridae/Begomovirus*	PCR / Direct Sanger Sequencing	Harimalala et al., 2015
			East African cassava mosaic Cameroon virus (EACMCV)	*Begomovirus manihotiscameroonense*			PCR / Direct Sanger Sequencing	
			East African cassava mosaic Kenya virus (EACMKV)	*Begomovirus manihotiskenyaense*			PCR / Direct Sanger Sequencing	
HAY1.3	Comoros/Havrara	15/09/2016	Cassava brown streak virus (CBSV)	*Ipomovirus brunusmanihotis*	ssRNA	*Potyviridae/Ipomovirus*	RT-PCR / Direct Sanger Sequencing	Azali et al., 2017
6 mois Blanc	Mayotte/Dembeni	26/03/2015	East African cassava mosaic virus (EACMV)	*Begomovirus manihotisafricaense*	ssDNA	*Geminiviridae/Begomovirus*	dsRNAs	Kwibuka et al., 2021
			Manihot esculenta-associated virus 1 (MEaV-1)	na	ssRNA	*Closteroviridae/Ampelovirus*		
			Manihot esculenta-associated virus 2 (MEaV-2)	na	ssRNA	*Closteroviridae/Ampelovirus*		
CRE11	Reunion Island/ Bassin Plat	25/02/2015	Manihot esculenta-associated virus 1 (MEaV-1)	na	ssRNA	*Closteroviridae/Ampelovirus*	dsRNAs	Kwibuka et al., 2021
HEL 3.1	Comoros/Helindje	15/09/2016	Ugandan cassava brown streak virus (UCBSV)	*Ipomovirus manihotis*	ssRNA	*Potyviridae/Ipomovirus*	RT-PCR / Direct Sanger Sequencing	Azali et al., 2017

### Molecular analysis of the cassava viromes

Total RNA was extracted using the RNeasy Plus Kit (Qiagen, Les Ulis, France) according to the manufacturer’s instructions. Total RNA quantity was assessed with the Qubit fluorometer (Thermo Fisher Scientific Inc., Waltham, MA) using the RNA HS Assay kit (Thermo Fisher Scientific, Illkirch, France).

**Fig. 1:**
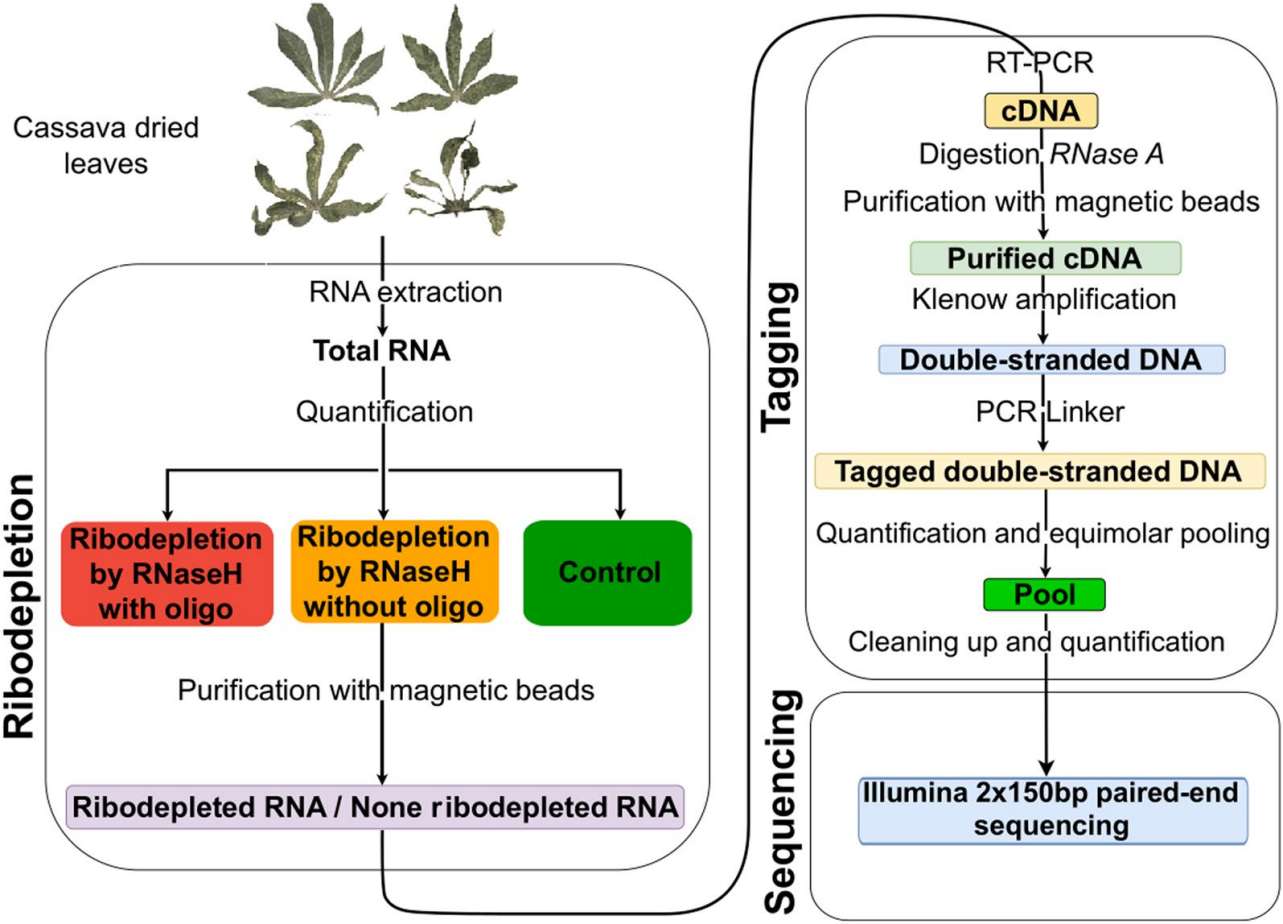
Schematic representation of the Ribodepletion-Multiplexing-Sequencing protocol with the ribodepletion, tagging and sequencing steps

A protocol for high-throughput sequencing based on ribodepletion of total RNA, dsDNA synthesis and tagging was implemented for cassava virome analysis (Fig. 1). Ribodepletion was achieved via cleavage of rRNA hybridised with specific DNA probes using RNaseH [25]⁠. A total of 273 DNA oligomers were designed on the basis of rRNA cassava sequences of reference cassava genome v8.1 (GCF_001659605.2). The oligomers were designed as described by Phelps et al*.* [25]⁠ using the Oligo-ASST Web tool (https://mtleelab.pitt.edu/oligo), resulting in a pool of 273 unique oligomers. Ribodepletion by RNaseH was performed as described by Phelps et al. [25]⁠ with slight modifications: the total amount of RNA per sample was reduced to 100 ng and the final concentration of oligomers was 0.036 µM. The RNA–DNA hybrids were digested using 10 U of thermostable RNase H (EURx, Gdańsk, Poland) at 65 °C for 10 min in a 20 µL volume. After digestion, the sample was purified using Mag-Bind total pure next-generation sequencing (NGS) beads (1.8X, Omega Bio-Tek, Tebubio, Le Perray en Yvelines, France) and ribodepleted RNAs were eluted in 35 µL of nuclease-free water. Two control treatments were used: the first consisted of total RNA direct use without any ribodepletion treatment and the second consisted of total RNA treated using RNaseH but in the absence of of rRNA specific complementary oligomers. Whereas the first control treatment was applied to every samples, this second control treatment was applied to the healthy cassava control sample and the 6 mois Blanc sample (Table 1). A total of 14 sample-treatment combinations was analysed.

Purified ribodepleted RNA was used for complementary DNA (cDNA) synthesis and tagging as described by François et al. [26]⁠, except purification which was done with Mag-Bind total pure next-generation sequencing (NGS) beads (1.8X, Omega Bio-Tek, Tebubio, Le Perray en Yvelines, France). Using that protocol, DNA amplicon sets with unique tags of 24 nt on both extremities are obtained (see François et al. [26] for details on tag sequences). Each sample was treated in triplicate with three different tags from the 96. Amplicons obtained were quantified using the Qubit dsDNA HS Assay Kit (Thermo Fisher Scientific, Illkirch, France) before equimolar pooling. The amplicon pool was then cleaned up using Mag-Bind total pure next-generation sequencing (NGS) beads (0.65X), and quantified using Qubit dsDNA HS Assay Kit. The pool was sent for 2 × 150 bp paired-end sequencing on an Illumina NovaSeq 6000 sequencer at Eurofins Genomics (Ebersberg, Germany). Amplicon pool was checked using High Sensitivity D5000 ScreenTape for Agilent Tapestation (Additional Fig. 1). Illumina sequencing library was constructed by the manufacturer with their PCR-based protocol. A 10% PhiX spike-in was used during sequencing.

### Bioinformatics analysis

After Illumina sequencing, reads were demultiplexed and the 24 nt tags were removed using Cutadapt v3.5 [27]⁠. The double indexed reads were quality controlled using Trimmomatic v0.35 [28]⁠, over a sliding window of five bases with an average quality of 20. Adapters were removed, and poor quality and/or short reads (fewer than 100 bases) were discarded. The cleaned reads were then used for similarity searches against a database of virus sequences from NCBI RefSeq (obtained in October 2022, release 213) and the cassava reference genome with MMseqs2 [29]⁠. The total number of reads assigned to the cassava genome, rRNA, and viruses were recorded. On a per sample basis, reads were de novo assembled using SPAdes v3.13.0 [30]⁠ and mapped back against the assembled contigs using bwa-mem2 v2.2.1 [31]⁠. Mapping statistics were determined using SAMtools v1.18 [32]⁠. The contigs and unmapped reads were then used in similarity searches against the above mentioned database using MMseqs2. For sequences identified as viruses, a second similarity search analysis was performed using BLASTn and BLASTx against the RefSeq viral database using an E-value of 10^–4^ as the cut-off threshold value for significant hits.

Viral contigs of more than 500 nucleotides (nt) were sorted by virus family before being aligned using MAFFT v7.453 [33]⁠ against representative genomes of this family obtained from GenBank in August 2023. Maximum-likelihood phylogenetic trees were inferred with FastTree v2.3 [34]⁠ using the general time reversible and gamma parameters. Branch supports were tested using the Shimodaira–Hasegawa procedure. Phylogenetic trees were edited using the ape R package [35]⁠.

In order to estimate the coverage of the largest viral contigs in relation to the number of sequenced reads per sample, sub-samplings of the contigs coverage data were performed. To this end, the actual number of reads mapped per position of contigs representing full size or nearly full size of viral genomes were sub-sampled 100 times for sets of decreasing sequencing efforts. Sequencing depth (the number of times a position was covered with a read) and breadth of the coverage (the proportion of the genome covered with a read) were calculated for each subsample.

## Results and discussion

### Effectiveness of ribodepletion

After demultiplexing raw reads, it was apparent that a large fraction of the reads (47%) presented with mismatching tags or had at least one of the two reads without identifiable tag (5%). Probable high index-switching rates associated to the use of PCR for sequencing library construction might be at the root of such issue. Comparable results were reported in other studies using similar library construction and sequencing procedure [36–38]. The index switching are known to results from to the formation of chimera during bulk amplification of tagged amplicons during library index PCR [36]. The use of PCR during library preparation from amplicons should thus be avoided for better results. In order to lie on the side of conservatism, we choose to only consider for further analysis the fraction of reads pairs that presented with matching tags (48% of the raw reads). After quality control of pairs with matching tags, 75% of the demultiplexed reads remained and the final number of reads associated to each of the studied sample/treatment combination varied from 3.9 to 21.9 million with a mean of 10.3 million.

**Fig. 2:**
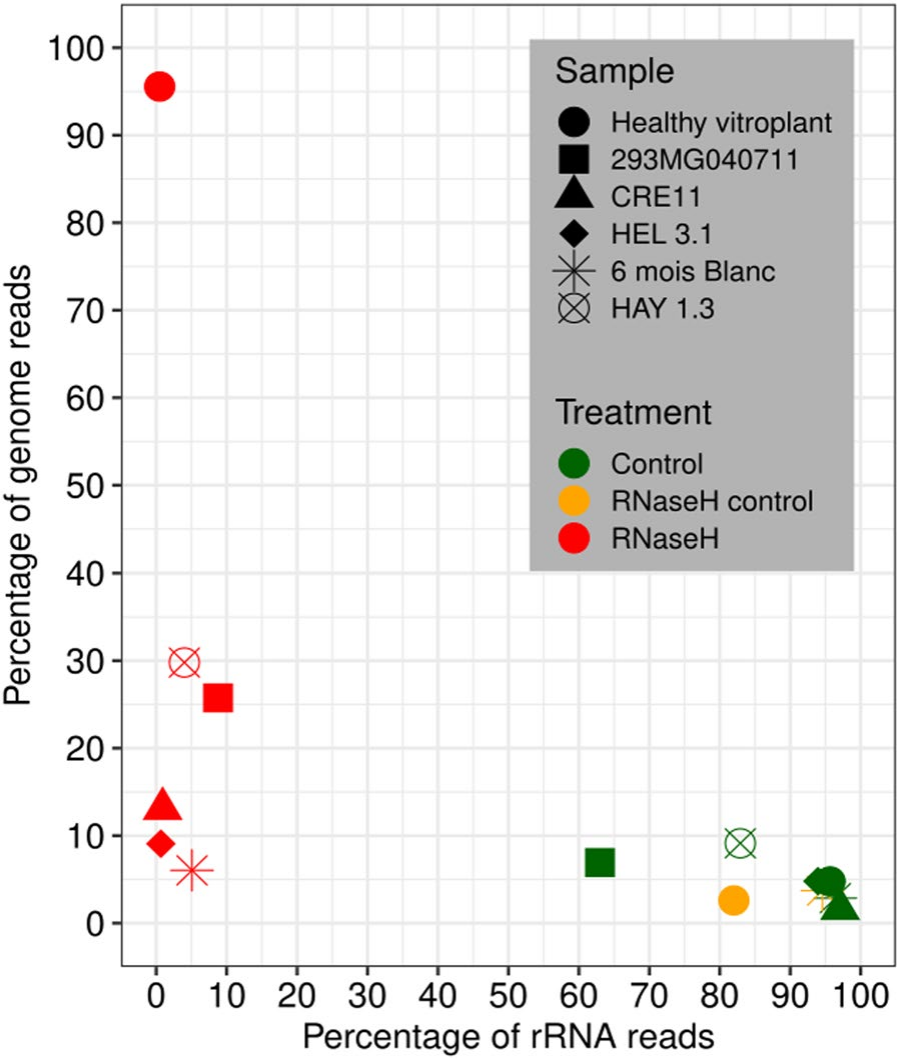
Percentage of reads assigned to cassava ribosomal RNAs (x-axis) and other cassava genome reads (y-axis) for each sample under the different treatments applied, as per key at the top right of the figure

In order to evaluate the effectiveness of the ribodepletion, the clean reads were used for an initial global classification (Fig. 2). Reads were classified as either cassava rRNA sequences, cassava genomic sequences or virus sequences. For the healthy vitroplant control without ribodepletion, the percentage of reads associated with rRNAs and cassava genome were 95.7% and 4.8%, respectively. These proportions were largely similar (82.0% and 2.6% for rRNA and genome, respectively) for the second control with RNaseH treatment but without probes. Conversely, after RNaseH treament, the percentage of reads from the rRNA and cassava genome were 0.5% and 95.6% respectively, indicative of a near-complete rRNA depletion. Similar trends were obtained for the other samples with a large decrease of rRNA reads after RNaseH treatments in comparison to the control without treatment or RNaseH treatment without probes. While proportions of cassava genome sequences and rRNA ranged from 1.6 to 9.1% and 63.0 to 97.1% respectively for controls, no samples gave more than 8.8% of rRNA reads after RNaseH treatment. However, the proportion of reads attributed to the cassava genome increased after ribodepletion, ranging from 6.0 to 29.8% (average 16.7%). The remaining sequences were unclassified (65.4% to 90.2%). It must be noted that such a large proportion of unclassified sequences was not observed for the healthy cassava control (mean: 93.7% of classified reads). Further attempts to classify these reads revealed hits with significant proportions for fungal RNAs and rRNAs (data not shown). Whereas the ribodepletion protocol presented here ensures efficient plant rRNA removal from total RNA as showed in previous studies [21, 25]⁠, our results also highlighted the importance of sample conservation and the limitations of using relatively old samples. Although we were able to extract and sequence RNA from dehydrated samples conserved at room temperature for up to eleven years, a large fraction of fungal RNA was obtained from the samples despite the absence of visible fungi growth.

### Estimation of the background

Estimating the proportion of viral reads from the negative control requires estimating the mean background contamination [39]⁠. Analysis of the ~ 7.6 M reads obtained after quality control of the negative control allowed us to assign 30 reads to viral genomic sequences, with a maximum of 20 reads to members of the *Potyviridae* family (Table 2). This represented less than four viral reads per million sequenced reads (less than three for members of *Potyviridae*). Note that establishing an exact threshold to determine positivity is not an easy feat using NGS data and more controls are required for a thorough statistical estimation of this threshold [39]⁠. A negative control made of healthy cassava herbarium in addition to the fresh cassava control would certainly have proved informative. However, based on the above estimation of the number of viral reads detected from the negative control, a conservative value of 100 reads per million sequenced reads (1 in 10,000 or 0.01%) would be used to filter our results, a threshold in line with reports from positive samples analysed using a similar approach [16, 40–42]⁠.

**Table 2:** Classification of viral sequence at the family level for each cassava sample after ribodepletion

Sample	Total number of reads	*Closteroviridae* members*	*Geminiviridae* members*	*Potyviridae* members*	Total number of viral reads*
Healthy vitroplant	7,637,666	1 (0)	9 (1)	20 (3)	30 (4)
293MG040711	9,693,254	460 (47)	2311 (239)	0 (0)	2771 (286)
HAY 1.3	4,530,258	492 (109)	111 (25)	4673 (1032)	5276 (1166)
6 mois Blanc	7,477,360	22 (3)	290 (39)	5 (1)	317 (43)
CRE11	11,705,482	48 (4)	93 (8)	1 (0)	142 (12)
HEL 3.1	4,456,320	0 (0)	12 (3)	8 (2)	20 (5)

^*^Rounded number of reads per million sequenced reads are indicated in parenthesis

### Taxonomic assignments and characterisation of plant viruses

Congruent with our background knowledge of the viruses infecting the tested samples (Table 1), reads were mainly assigned to viruses of the *Closteroviridae* (ssRNA +), *Geminiviridae* (circular ssDNA), and *Potyviridae* (ssRNA +) families (Table 2). For sample 293MG040711 infected by three begomoviruses (African cassava mosaic virus, ACMV; East African cassava mosaic Cameroon virus, EACMCV and East African cassava mosaic Kenya virus, EACMKV), the presence of the begomoviruses previously characterised using the RCA-RFLP method [23] was confirmed. A total of 2,311 begomovirus reads were detected (513 ACMV reads, 1,529 EACMCV reads and 233 EACMKV reads). In addition to virus detection, we also obtained contigs of ACMV (176 to 1,146 nt), EACMCV (173 to 2,698 nt) and EACMKV (456 to 1,244 nt). Five contigs of more than 500 nt were used for phylogenetic inference. These contigs were clustered (with nucleotide identities ranging from 94.2 to 100%) with sequences of other isolates obtained from Madagascar (Additional Fig. 2). Unexpectedly, 443 reads of Manihot esculenta associated ampelovirus 2 (genus *Ampelovirus*, assembled in ten contigs of 238 to 1,830 nt) were also obtained from the sample. The contigs clustered with isolates of Manihot esculenta-associated virus (Additional Fig. 3), which was also identified in Madagascar. It is important to notice that previous analyses of the sample focused on CMGs and no ampelovirus indexing was thus carried out. Besides highlighting the diversity and distribution of the cassava ampeloviruses, this also demonstrates that the NGS protocol used is suitable for the co-detection of RNA and DNA viruses.

For the HAY1.3 sample, the CBSV (genus *Ipomovirus*) was previously detected by RT-PCR (Table 1). This detection was confirmed in our analysis, with a total of 4,673 reads assigned to this species. These reads were assembled into three CBSV contigs including one of 8,582 nt, almost the entire length of the closest isolate whose full genome is available (MK103393; 9,002 nt). The phylogeny of the CBSV (Additional Fig. 4) revealed that the contig was closely related (maximum nucleotide identity 95.8%) to three other isolates obtained from samples collected in Grande Comore [24]⁠. Finally, as for sample 293MG040711, unexpected ampelovirus reads were obtained from sample HAY1.3 (N = 459) and six contigs of more than 500 nt were assembled. The associated phylogeny shows that these contigs were most closely related to other isolates of Manihot esculenta-associated ampelovirus 1 from Madagascar and Mayotte [9]⁠. The details of the contigs are presented in Additional Table 1.

### The importance of sample preservation for virus detection

For 6 mois Blanc sample, from which sequences of ampeloviruses and begomoviruses had previously been obtained, we could only confidently confirm the detection of begomoviruses with 272 reads. However, no medium size contigs could be assembled and no further classification were attempted. The last two samples, CRE11 and HEL3.1, while giving some virus reads, had counts of similar magnitude as the healthy control and as such were not considered for further analysis. We were thus unable to confirm the previous viral identification for these three samples. The fact that these three samples had the lowest proportion of classified reads (maximum of 14% in comparison to ~ 34% for both 293MG040711 and HAY1.3) points again to the importance of sample preservation for accurate analysis, most importantly when dealing with low titer viruses that may be difficult to detect [43, 44]. Our samples were collected between 2011 and 2016 and were preserved in envelopes in a herbarium. High susceptibility of RNA to hydrolytic attack [45]⁠ and long-term storage of dried leaves, known to be associated to damage of nucleic acids [46]⁠, might have had a negative impact on virus identification [47]⁠. Comprehensive RNA quality control would thus be recommanded before using the described protocol.

### Influence of sequencing depth on viral genome coverage

**Fig. 3:**
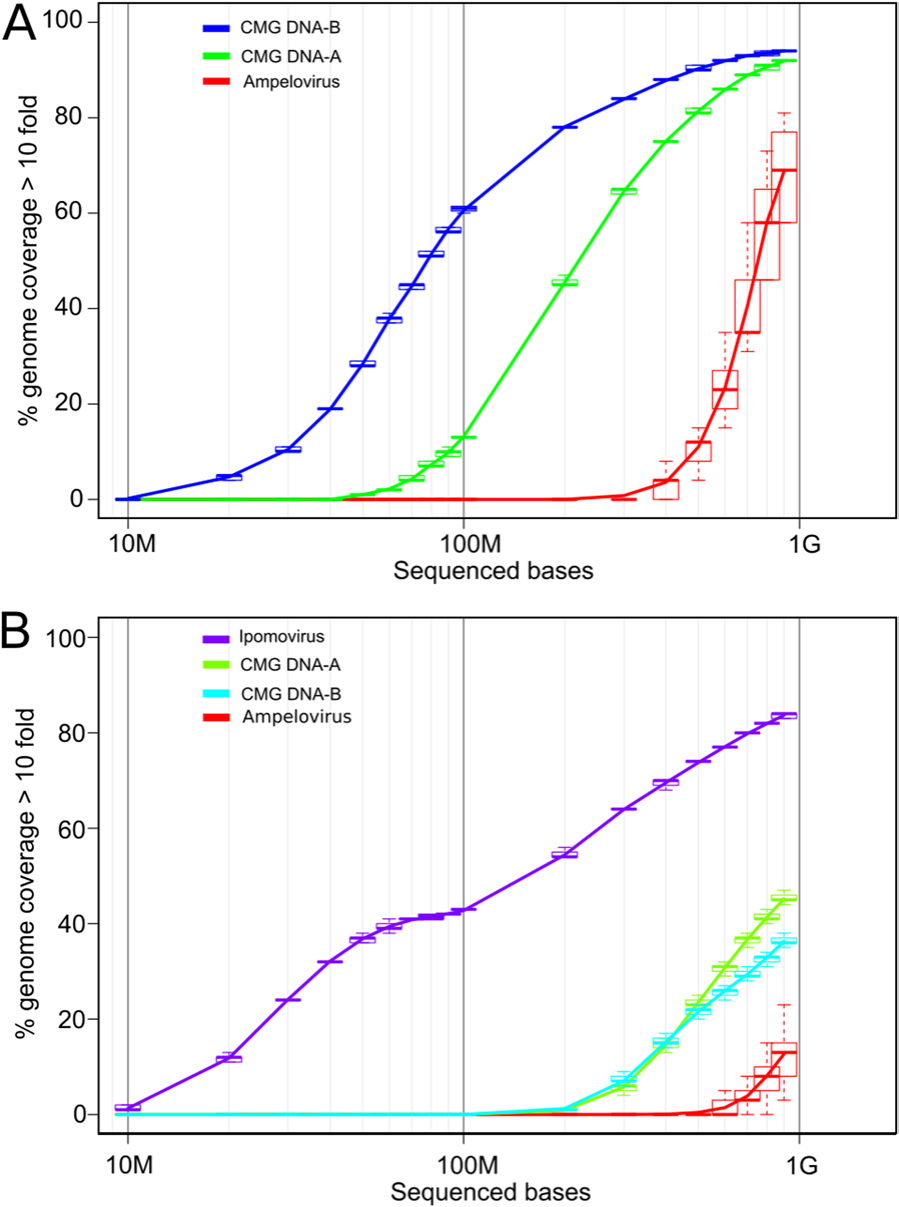
Intrapolation of the breadth of coverage at a 10X coverage (y-axis) for representative genomes of ampeloviruses, cassava geminiviruses DNA-A and DNA-B components and ipomoviruses according to the number of sequenced bases (x-axis, in Log10 scale) for sample 293MG040711 (**A**) and HAY 1.3 (**B**)

In order to evaluate the sequencing effort required for virus characterisation, we choose to thoroughly sequence each sample to later estimate the actual number of samples that could be multiplexed while maintaining the ability to identify the viruses in these samples. For samples 293MG040711 and HAY1.3, the breath of coverage (i.e. the proportion of the viral genome that is covered with reads) was calculated at a sequencing depth of 10X (i.e. meaning that a given position has to be covered with at least ten reads to be considered) for sets of subsampled reads. We obtained the distribution of coverage percentage of the genome for each species of virus depending on the number of sequenced bases (Fig. 3). For sample 293MG040711, the breadth of coverage of CMGs DNA-A and DNA-B components were both above 90% and for the ampelovirus genome this figure was 88% (Fig. 3A). For sample HAY 1.3, the breadth of coverage was 46%, 37% and 28% for CMGs DNA-A, CMGs DNA-B and ampelovirus genomes, respectively. It was 84% for the CBSV genome (Fig. 3B). Not all the viruses benefited from the same efficiency of characterisation; these differences could be attributed to variations in abundance [48, 49]⁠ and/or variations in RNA stability [50]⁠. As we were not able to obtain full genome 10X coverage for any of the analysed viruses, the significance of the results remain limited. However, for CMGs DNA-A and DNA-B sequences from 293MG040711, the curves tended to plateau, indicating that 100% breadth of coverage may not be achievable for these viruses. Conversely, steady increases in breadth of coverage were observed for the ampelovirus genome from 293MG040711 and for all viruses identified from the HAY1.3 sample. This latter observation indicates that the addition of new reads would improve virome characterisation. As such, any increases in the number of multiplexed samples, thus reducing the per-sample read numbers, would decrease our ability to characterize viral genomes. The multiplexing/coverage trade-off is delicate and depends on the scientific goal of the experiment. For virus detection, without any a priori, the sequencing effort in this study was sufficient to improve on previous knowledge of the virome of some samples. However, for poorer quality samples, analysis was unsuccessful. The poor quality of the samples that we analysed limited the sequencing quality, resulting in, at best, only a third of the sequences being successfully catalogued. Given that for the healthy cassava control, obtained from fresh material, 84% of the total reads were classified, a three-fold increase in usable reads would be expected in virome characterisation, if fresh samples were used. This would convert to ~ 42 samples analysed in a run (14 combinations of samples and treatments were analysed here) that could conveniently be limited to 32 to treat samples in triplicates and employ a 96-tag scheme.

## Conclusion

The originality of the procedure lies in the combination of two widely used protocols for ribodepletion and amplicon tagging in order to make virus detection from total RNA extracts more affordable. Whereas our work demonstrates that ribodepletion with RNaseH effectively removed most rRNA from total cassava RNA, our results also point to the importance of sample conservation for effective ribodepletion and virus detection. The strategy made it possible to detect RNA and DNA viruses and obtain contigs with near full-length viral genomes of target viruses. Although specific probe design has to be conducted depending on the plant species analysed, the procedure remains an inexpensive alternative that can be adapted to any plant whose rRNA sequences are known. With a per-sample ribodepletion and tagging price of around 18€, cost savings are achievable on both ribodepletion and multiplexing. The ability to multiplex up to 32 samples in a single library before sequencing in a single lane makes this an attractive alternative method of virus detection and characterisation for research studies in plant virus epidemiology.

## Supplementary Information

Additional file 1.

Additional file 2.

Additional file 3.

Additional file 4.

Additional file 5.

## Data Availability

Sequence data used and analysed during the current study are available at the NCBI Short read archive under the BioProject PRJNA1174894.

## Abbreviations

ACMBFV: African cassava mosaic Burkina Faso virus
ACMV: African cassava mosaic virus
CBSD: Cassava Brown Streak Disease
CBSV: Cassava brown streak virus
CMD: Cassava Mosaic Disease
CMGs: Cassava mosaic Geminiviruses
CMMGV: Cassava mosaic Madagascar virus
dsRNA: Double-stranded RNA
EACMCV: East African cassava mosaic Cameroon virus
EACMKV: East African cassava mosaic Kenya virus
EACMMV: East African cassava mosaic Malawi virus
EACMV: East African cassava mosaic virus
EACMZV: East African cassava mosaic Zanzibar virus
GLRaV1: Grapevine leafroll-associated virus 1
GLRaV13: Grapevine leafroll-associated virus 13
GLRaV3: Grapevine leafroll-associated virus 3
GLRaV4: Grapevine leafroll-associated virus 4
ICMV: Indian cassava mosaic virus
LChV2: Little cherry virus 2
MEaV: Manihot esculenta-associated ampelovirus
NGS: Next Generation Sequencing
PAVA: Pistachio ampelovirus A
PBNSPaV: Plum bark necrosis stem pitting-associated virus
PMWaV1: Pineapple mealybug wilt-associated virus 1
PMWaV2: Pineapple mealybug wilt-associated virus 2
PMWaV3: Pineapple mealybug wilt-associated virus 3
RCA-RFLP: Rolling Circle Amplification-restriction fragment length polymorphism
Ribo-M-Seq: Ribodepletion-Mutliplexing-Sequencing
rRNA: Ribosomal RNA
SACMV: South African cassava mosaic virus
siRNA: Small interfering RNA
SLCMV: Sri Lankan cassava mosaic virus
circular ssDNA: Circular single-stranded DNA
ssDNA: Single-stranded DNA
ssRNA: Single-stranded RNA
UCBSV: Uganda cassava brown streak virus
VANA: Virion Associated Nucleic Acid
YaV1: Yam asymptomatic virus 1
