# Supplementary figures and images for: A ribodepletion and tagging protocol to multiplex samples for RNA-seq based virus detection: application to the cassava virome
Source: Virol J. 2025 Feb 5;22:27. doi: 10.1186/s12985-025-02634-9 (PMC11796006; doi:10.1186/s12985-025-02634-9)

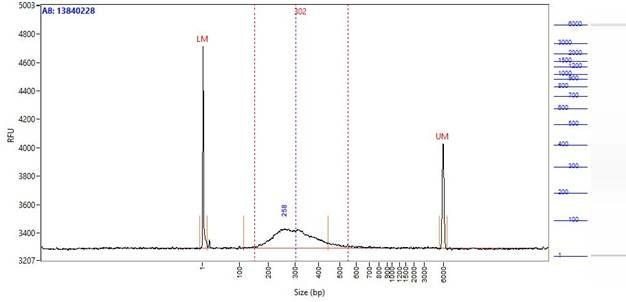

Supplement: Supplementary file 1 — Additional file 1. [file 12985_2025_2634_MOESM1_ESM.tif]

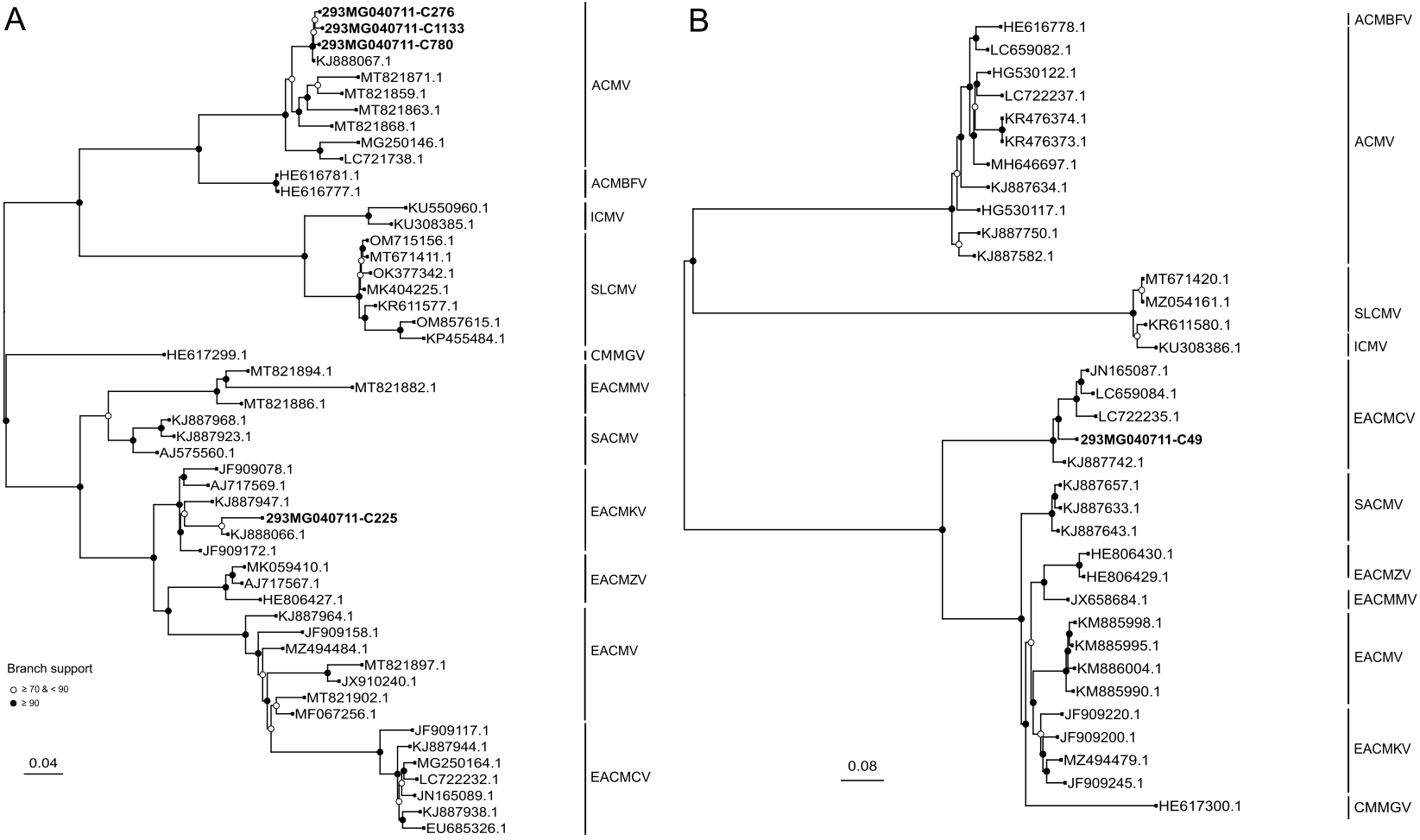

Supplement: Supplementary file 2 — Additional file 2. [file 12985_2025_2634_MOESM2_ESM.tif]

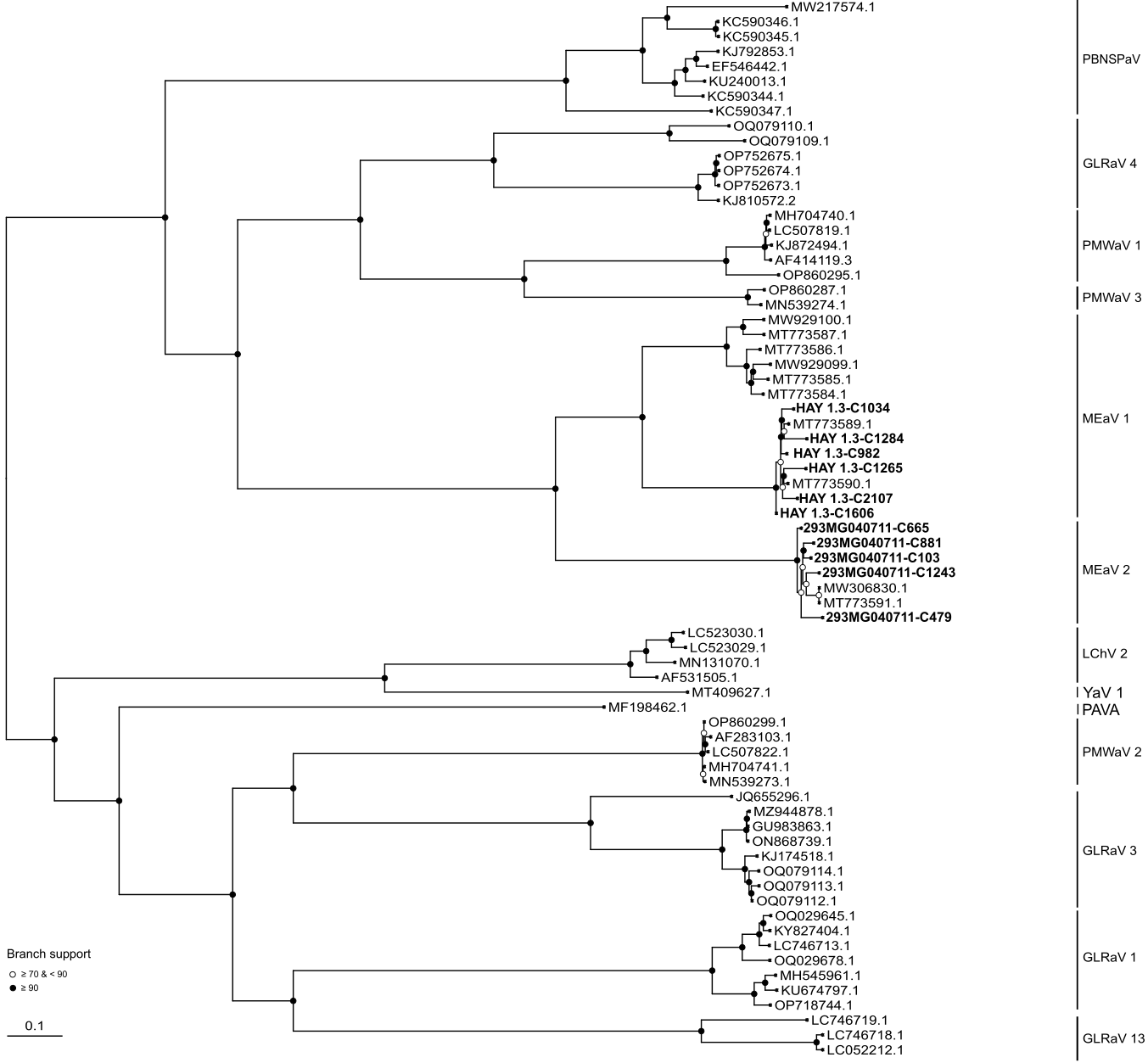

Supplement: Supplementary file 3 — Additional file 3. [file 12985_2025_2634_MOESM3_ESM.tif]

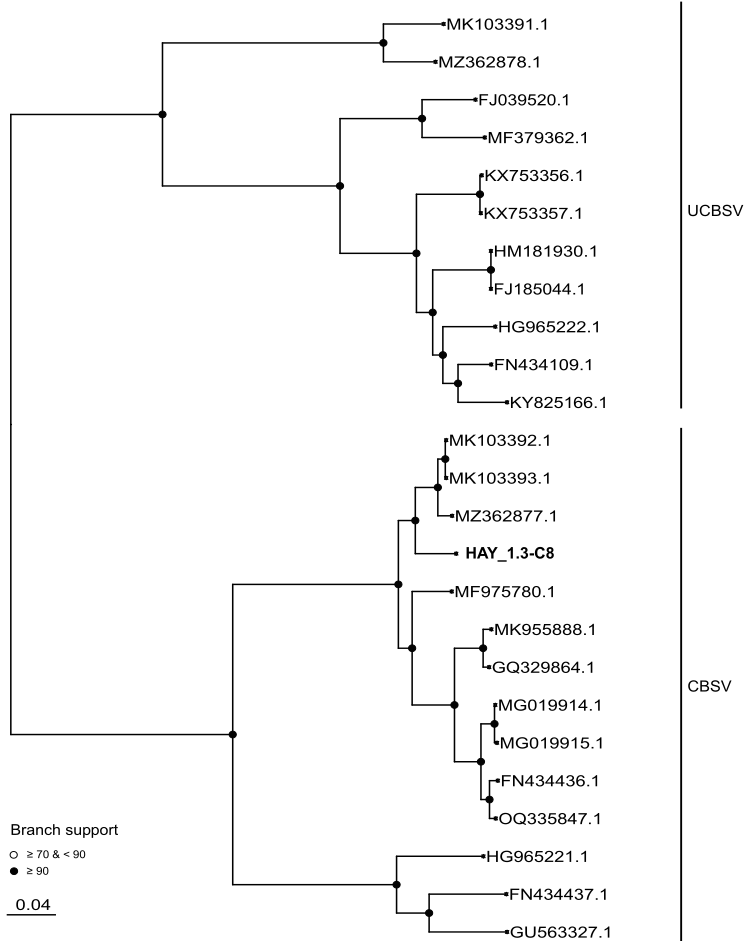

Supplement: Supplementary file 4 — Additional file 4. [file 12985_2025_2634_MOESM4_ESM.tif]
